# Supplementary material for: Surface uplift in the Central Andes driven by growth of the Altiplano Puna Magma Body
Source: Nat Commun. 2016 Oct 25;7:13185. doi: 10.1038/ncomms13185 (PMC5093326; doi:10.1038/ncomms13185)
Supplement: Supplementary Information — Supplementary Figures 1-2, Supplementary Tables 1-2 and Supplementary References. [file ncomms13185-s1.pdf]

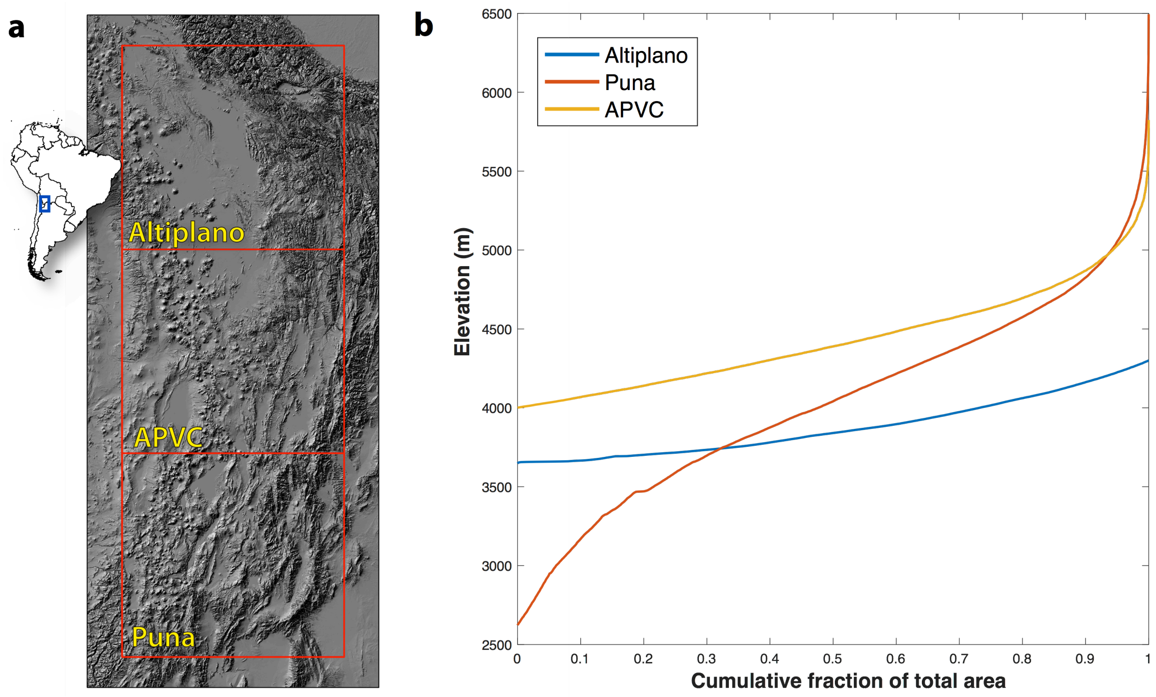

Supplementary Figure 1. Cumulative distributions of elevation for the Altiplano, Puna, and Altiplano-Puna Volcanic Complex (APVC) plateaus. **a** is a map showing the boxes delineating the three primary plateaus of the Altiplano-Puna. Each box was approximately equal in area, however the lower bound of the Puna box and upper bound of the Altiplano box are not shown here. **b** shows the cumulative elevation distributions for each box. Data used for this analysis come from the 1 arc-minute ETOPO1 elevation dataset. The Altiplano plateau has the narrowest range of elevation, and the lowest mean elevation. The Puna plateau has a higher mean and the greatest elevation range. The S shape of the distribution highlights the basin-and-range architecture of the eastern Puna. The APVC has the highest mean elevation of the three plateaus, and lacks the topographic relief that characterizes the Puna to the south. Both the APVC and the Puna share similarly high-elevation summits.

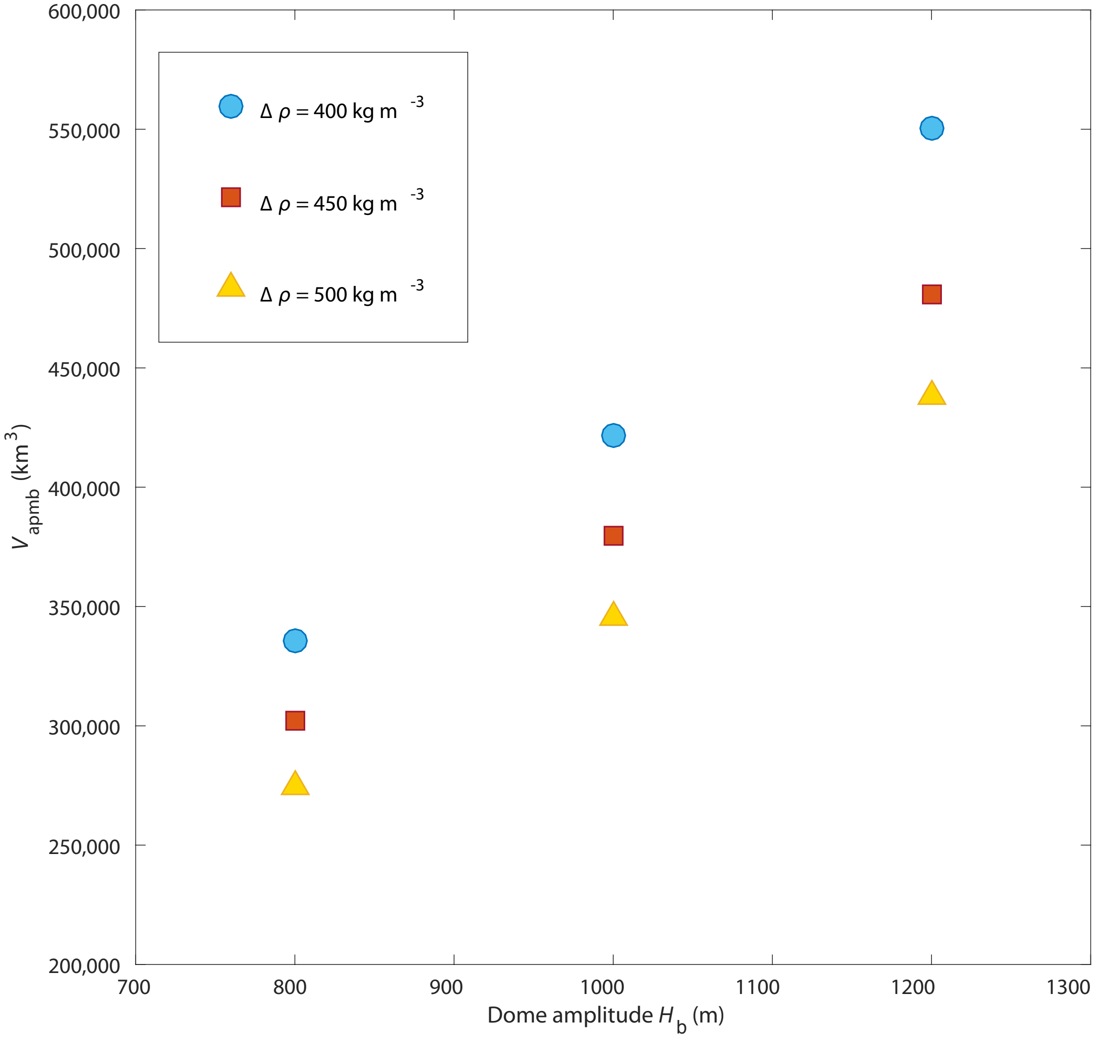

Supplementary Figure 2. Buried load isostatic model results showing calculated Altiplano Puna Magma Body (APMB) volume as a function of topographic dome amplitude. Results are plotted for a range of Δρ values. A greater density differential yields a smaller melt volume for a given height of topography.

Parameter		Units
Total volume of APVC	12800	**km^3**
Total area of APVC	55000	**km^2**
Area occupied by calderas	11000	**km^2**
Area outside of calderas	44000	**km^2**
Intra- caldera thickness	0.71	**km**
Caldera fill volume	7857	**km^3**
Outside fill volume	4943	**km^3**
Inter-caldera thickness	**0.11**	**km**

Supplementary Table 1. Estimates of ignimbrite thickness outside of calderas on the APVC. Ignimbrite volume and intra-caldera thickness data are taken from Salisbury et al., 2011[12].

Symbol	Name	Value	Units
g	Gravity	9.81	m s^{-2}
H_b	Topographic amplitude	800-1200	m
W_i	Buried load root profile	-	m
W_{ivol}	Buried load root volume		km^3
Fwhm	Full width at half max	232	km
V_{apmb}	Modeled APMB volume	*see Table 2*	km^3
ρ_c	Density of crust	2700-2850	kg m^{-3}
ρ_a	Density of asthenosphere	3200-3300	kg m^{-3}
k	wave number	-	m^{-1}
Te	Elastic thickness	15000	m
E	Young's Modulus	70	Gpa
ν	Poisson's Ratio	0.25	-
D	Flexural Rigidity	$2x10^{22}$	N m
α	Volcanic volume	15,000	km^3
β	Plutonic:volcanic	*see Table 2*	-
μ	crust:mantle provenance	0.5	-
η	dense residue:melt	0.5	-
λ	arc-parallel length	230	km
ξ	Mantle magma prod. rate	*see Table 2*	km^3 km^{-1} Myr^{-1}
τ	Timescale of melt accumulation	11	Myr

Supplementary Table 2. Parameters used in the isostatic and arc mantle magma production rate models.

Supplementary References

1. Salisbury, M. *et al.* $^{40}Ar/^{39}Ar$ chronostratigraphy of Altiplano-Puna volcanic complex ignimbrites reveals the development of a major magmatic province. *Geological Society of America Bulletin* **123,** 821–840 (2011).
